# Soil microbial responses to forest floor litter manipulation and nitrogen addition in a mixed-wood forest of northern China

**DOI:** 10.1038/srep19536

**Published:** 2016-01-14

**Authors:** Xiao-Lu Sun, Jing Zhao, Ye-Ming You, Osbert Jianxin Sun

**Affiliations:** 1 College of Forest Science, Beijing Forestry University, Beijing 100083, China; 2 Institute of Forestry and Climate Change Research, Beijing Forestry University, Beijing 100083, China

## Abstract

Changes in litterfall dynamics and soil properties due to anthropogenic or natural perturbations have important implications to soil carbon (C) and nutrient cycling via microbial pathway. Here we determine soil microbial responses to contrasting types of litter inputs (leaf vs. fine woody litter) and nitrogen (N) deposition by conducting a multi-year litter manipulation and N addition experiment in a mixed-wood forest. We found significantly higher soil organic C, total N, microbial biomass C (MBC) and N (MBN), microbial activity (MR), and activities of four soil extracellular enzymes, including β-glucosidase (BG), N-acetyl-β-glucosaminidase (NAG), phenol oxidase (PO), and peroxidase (PER), as well as greater total bacteria biomass and relative abundance of gram-negative bacteria (G-) community, in top soils of plots with presence of leaf litter than of those without litter or with presence of only fine woody litter. No apparent additive or interactive effects of N addition were observed in this study. The occurrence of more labile leaf litter stimulated G-, which may facilitate microbial community growth and soil C stabilization as inferred by findings in literature. A continued treatment with contrasting types of litter inputs is likely to result in divergence in soil microbial community structure and function.

Intensification of global change has profound impacts on the structure and function of forest ecosystems. Such events as windthrow and tree mortality as results of extreme climatic events, or anthropogenic N deposition and harvesting, can markedly modify the biotic and abiotic conditions on forest floor, thereby altering soil C and nutrient cycling via microbial pathway^1^,^2^. However, few studies have investigated how combined changes in forest floor litter dynamics and N deposition would affect soil processes. Forest floor litter consists of both nutrient-rich leaf litter and lignin-rich woody detritus. Shifts in the relative abundance of the two types of litter may lead to changes in soil microbial community structure, consequently affecting microbial regulation of soil C and nutrient processes through the linkage between microbial community structure and function^3^,^4^,^5^,^6^,^7^. While leaf litter is commonly found to be associated with abundance of diverse soil microbial communities^8^,^9^, the occurrence of woody litter tends to promote the abundance of saprophytic fungi specialized in lignin degradation^10^. N deposition, on the other hand, can either stimulate the growth of soil microbial community by ameliorating N-limitation and relieving C-limitation through enhancement of aboveground NPP and litter quality^1^,^11^, or impose negative impacts on soil microorganisms, especially the white-rot fungi, which is a major producer of soil oxidase, by increasing soil acidity or enriching toxic [NH_4_^+^]^2^,^12^,^13^. Moreover, excessive N leads to microbial C-limitation by increasing resistance to lignin degradation, decreasing plant belowground NPP or inhibiting soil microorganisms^11^,^13^. In labile substrate, N addition stimulates the growth of microbes and enzyme activities by alleviating microbial N-limitation; whereas in recalcitrant substrate, lignin-limitation caused by a reduction of N-suppressive phenol oxidase may restrain soil microbial community^14^,^15^,^16^,^17^,^18^,^19^.

Based on relevant findings in literature, we postulate that differential occurrence of leaf litter and woody litter on forest floor would lead to divergence in soil microbial community structure and function because of contrasting chemistry and recalcitrance, i.e. nutrient-rich vs. lignin-rich, with different levels of N deposition exacerbating the substrate-induced differences in soil microbial traits. To test this hypothesis, we conducted a multi-year litter manipulation and N addition experiment in a mixed pine (*Pinus tabulaeformis* Carr.) and oak (*Quercus wutaishanica* Mayr.) forest in temperate northern China. Replicated field plots were set up in 2009 with three rates of N addition (0, N_0_; 5 gN·m^−2^·a^−1^, N_5_; 10 g N·m^−2^·a^−1^, N_10_) and four types of litter placement (removal, Litt_nil_; leaf litter, Litt_leaf_; fine woody litter, Litt_woody_; mixture of leaf and fine woody litter, Litt_mix_), and selective soil physiochemical properties and microbial traits were studied four years after the initial treatments.

## Results

Both litter mass density and chemistry varied among plots of different types of litter placement. Litter mass density in the Litt_woody_ plots was less than half of that in the Litt_leaf_ and Litt_mix_ plots. The litter samples from the Litt_woody_ plots had significantly lower contents of N (%N) (F = 31.587, d.f. = 2, *p* < 0.001) and acid-unhydrolyzable residue (%AUR) (F = 14.882, d.f. = 2, *p* < 0.001), and significantly higher C:N ratio (F = 18.711, d.f. = 2, *p* < 0.001) and AUR:N ratio (F = 7.928, d.f. = 2, *p* = 0.001), than those from the Litt_leaf_ and Litt_mix_ plots (Table 1). The effects of the litter treatment were significant on both soil organic C (SOC) content (F = 11.182, d.f. = 3, *p* < 0.001) and total N (TN) content (F = 17.556, d.f. = 3, *p* < 0.001); values of SOC and TN were all higher in the Litt_mix_ and Litt_leaf_ plots than in the Litt_woody_ and Litt_nil_ plots. TN was also significantly affected by the level of N addition (F = 3.051, d.f. = 2, *p* = 0.050), and was highest in the N_5_ treatment and lowest in the N_0_ treatment (Table 2). Soil C:N ratio and soil pH were not significantly affected by the litter treatment and the level of N addition (Table 2).

**Figure 1:**
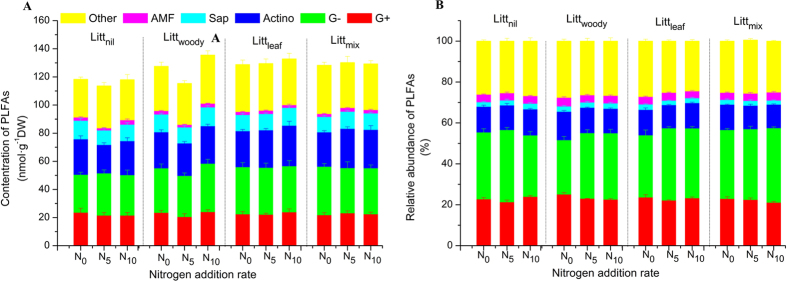
Soil microbial community structure based on PLFAs under different treatments of litter and nitrogen in a mixed pine/oak forest in central North China: (**A**) Concentrations; (**B**) Relative abundance. Treatments began in September 2009 and were applied annually; samples were collected in August 2014. Vertical bars indicate one standard error of means (n = 5). N_0_, zero rate of nitrogen addition; N_5_, nitrogen addition at 5 g∙m^−2^∙a^−1^; N_10_, nitrogen addition at 10 g∙m^−2^∙a^−1^; Litt_nil_, litter removal; Litt_woody_, fine woody litter; Litt_leaf_, leaf litter; Litt_mix_, mixed leaf and fine woody litter.

The soil microbial community composition in terms of PLFAs was significantly affected by the litter treatment (F = 3.274, d.f. = 1, *p* = 0.043) as tested by the PERMANOVA procedure, in particular the quantity of total bacteria (F = 2.934, d.f. = 3, *p* = 0.042) and the relative abundance of gram-negative bacteria, G^−^ (F = 3.371, d.f. = 3, *p* = 0.025). The quantity of total bacteria and the relative abundance of G^−^ were both higher in the Litt_mix_ and Litt_leaf_ plots than in the Litt_nil_ and Litt_woody_ plots (Fig. 1). The relative abundance of G^−^ was also significantly (F = 4.409, d.f. = 2, *p* = 0.023) affected by the level of N addition, and was higher in the N_5_ treatment than in the N_0_ treatment; whilst the effect of N addition on the concentration of total PLFAs was only marginal (F = 2.934, d.f. = 1, *p* = 0.051). The ratio of fungal to bacterial biomass (F:B ratio) was not significantly affected by either the litter treatment or the level of N addition, nor were there significant interactions between the litter treatment and the level of N addition in the effects on soil microbial community structure.

**Figure 2:**
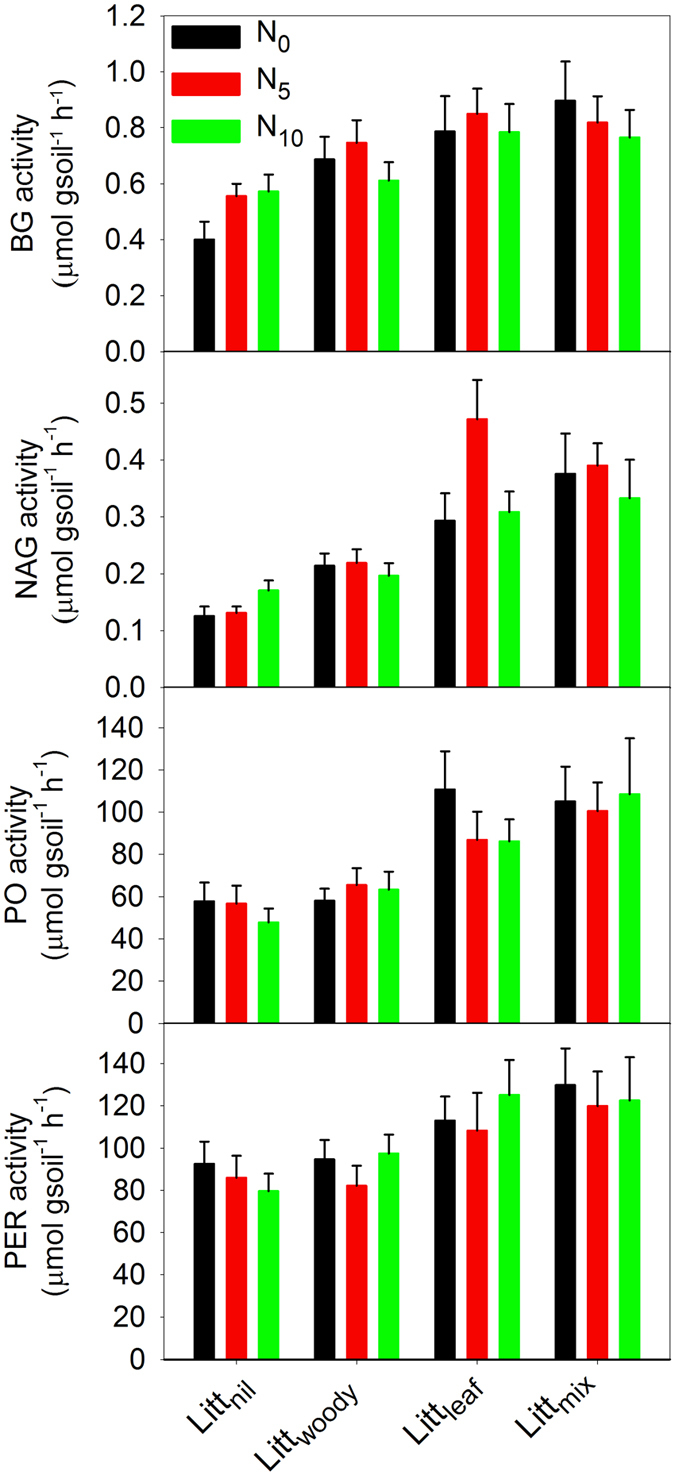
Differences in extracellular enzyme activities among treatments of litter and nitrogen in a mixed pine/oak forest in central North China. Treatments began in September 2009 and were applied annually. Vertical bars indicate one standard error of means (n = 5). BG, β-glucosidase; NAG, N-acetyl-β-glucosaminidase; PO, phenol oxidase; PER, peroxidase; N_0_, zero rate of nitrogen addition; N_5_, nitrogen addition at 5 g∙m^−2^∙a^−1^; N_10_, nitrogen addition at 10 g∙m^−2^∙a^−1^; Litt_nil_, litter removal; Litt_woody_, fine woody litter; Litt_leaf_, leaf litter; Litt_mix_, mixed leaf and fine woody litter.

**Figure 3:**
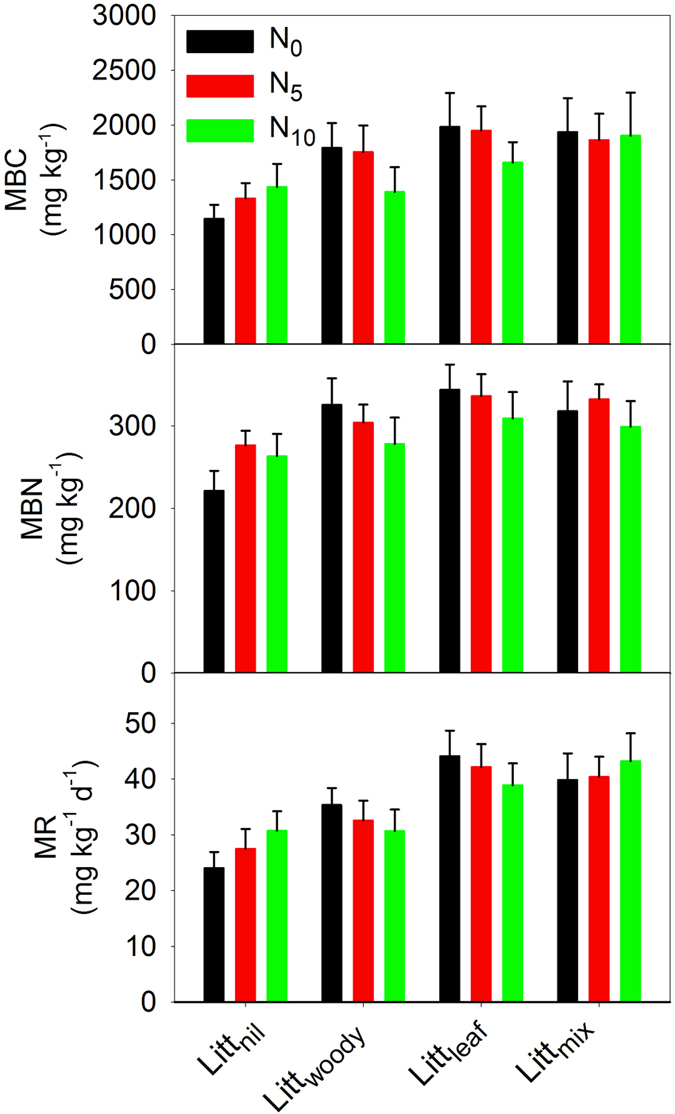
Differences in microbial biomass carbon (MBC), nitrogen (MBN) and microbial activity (MR) among treatments of litter and nitrogen in a mixed pine/oak forest in central North China. Treatments began in September 2009 and were applied annually. Vertical bars indicate one standard error of means (n = 5). N_0_, zero rate of nitrogen addition; N_5_, nitrogen addition at 5 g∙m^−2^∙a^−1^; N_10_, nitrogen addition at 10 g∙m^−2^∙a^−1^; Litt_nil_, litter removal; Litt_woody_, fine woody litter; Litt_leaf_, leaf litter; Litt_mix_, mixed leaf and fine woody litter.

**Figure 4:**
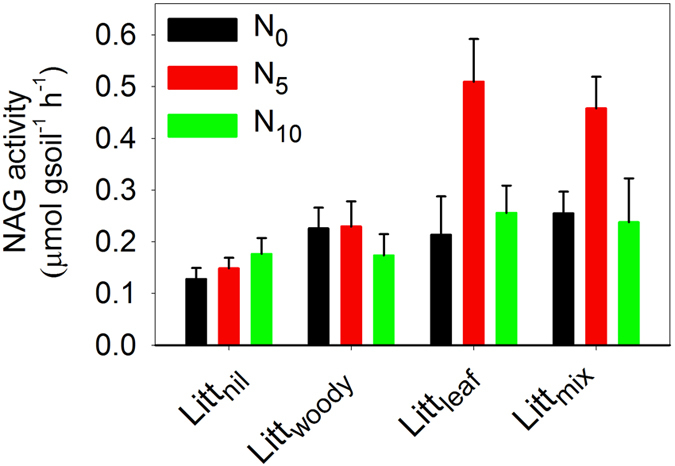
Differences in activity of N-acetyl-β-glucosaminidase (NAG) among treatments of litter and nitrogen sampled in October 2014 in a mixed pine/oak forest in central North China. Treatments began in September 2009 and were applied annually. Vertical bars indicate one standard error of means (n = 5). N_0_, zero rate of nitrogen addition; N_5_, nitrogen addition at 5 g∙m^−2^∙a^−1^; N_10_, nitrogen addition at 10 g∙m^−2^∙a^−1^; Litt_nil_, litter removal; Litt_woody_, fine woody litter; Litt_leaf_, leaf litter; Litt_mix_, mixed leaf and fine woody litter.

The activities of four extracellular enzymes studied, including BG, NAG, PO, and PER, were all significantly affected by the litter treatment as tested by repeated measures ANOVA acoss sampling times (BG: F = 4.208, *p* = 0.010; NAG: F = 15.90, *p* < 0.001; PO: F = 5.727, *p* = 0.002; PER: F = 3.270, *p* = 0.030; d.f. = 3 for all) (Table 3); they were mostly higher in the Litt_mix_ and Litt_leaf_ plots than in the Litt_woody_ and Litt_nil_ plots (Fig. 2). MBC, MBN and MR were also significantly affected by the litter treatment (MBC: F = 6.349, *p* = 0.001; MBN: F = 3.506, *p* = 0.022; MR: F = 9.155, *p* < 0.001; d.f. = 3 for all) (Table 3); they were mostly higher in the Litt_mix_ and Litt_leaf_ plots than in the Litt_woody_ and Litt_nil_ plots (Fig. 3). There was a significant interactive effect on the NAG activity between the litter treatment and the level of N addition when sampled in October 2014 (F = 2.306, d.f. = 6, *p* = 0.049) (Table 3); the NAG activity in the Litt_leaf_ and Litt_mix_ plots of the N_5_ treatment was markedly greater than in other treatments (Fig. 4).

## Discussion

In this study, we found that the microbial traits in the top soils of a mixed-wood forest in temperate northern China are mainly differentiated by the occurrence of leaf litter, without apparent additive or interactive effects of N addition. In the experimental plots where leaf litter are present (i.e. in Litt_leaf_ and Litt_mix_ plots), the top soils had greater activities of BG, NAG, PO, and PER, which are soil extracellular enzymes directly involved in C metabolisms, and greater microbial biomass C, microbial biomass N and microbial activity, corresponding to greater litter mass density and lower values of C:N and AUR:N ratios, than in the plots where leaf litter are removed (i.e. in Litt_nil_ and Litt_woody_ plots). This is because that nutrient-rich leaf litter is more readily degradable than lignin-rich woody litter^14^, and that exclusion of leaf litter would constrain soil microbial growth and function by imposing energy limitation. Varying findings have been reported regarding the differential effects of leaf litter and woody litter on soil microbial characteristics^20^,^21^,^22^. Changes in soil microbial traits in response to contrasting types of litter inputs were also found to be associated with significant changes in soil organic C and total N, signifying the importance of leaf litter as a source of soil organic matter in top soils and the underlying controls of microbial communities in soil C and N processes.

Examination of the microbial community structure using PLFAs revealed that the total biomass of bacteria was higher in soils with presence of leaf litter than in soils with presence of woody litter, in agreement with findings from other studies that bacteria are adapted to utilize easily decomposed C sources (more cellulose, less lignin)^7^,^13^,^23^. Moreover, the relative abundance of gram-negative bacteria was significantly higher with presence of leaf litter, indicating that the occurrence of more labile leaf litter stimulated gram-negative bacteria, which is a specific group of bacteria often classified to the copiotrophic community and is activated by high C and nutrient availability^3^,^4^. It is known that the extracellular enzymes such as BG are produced by gram-negative bacteria and are cell-bounded, giving them advantages in avoiding diffusive loss and enhancing resources utilization^4^,^24^. Our results suggest that the presence of leaf litter increases production of cellulosic hydrolases and lignin oxidase as compared with occurrence of woody litter, facilitating microbial community growth and soil C stabilization as inferred by findings in literature^6^. It can be predicted that a continued treatment with contrasting types of litter inputs would result in divergence in soil microbial community structure and function.

Many previous studies have established that, in labile substrate, N addition stimulates the growth of microbes and enzyme activities by alleviating microbial N-limitation^14^,^15^,^16^,^17^,^18^,^19^. In this study, we found that a moderate N addition at 5 gN·m^−2^·a^−1^ markedly increased the activity of N-acetyl-β-glucosaminidase.

## Methods

### Study site

The study is located in Ling Kong Mountain (latitude 36°38.736′N and longitude 112°06.967′E) of Shanxi province, northern China. The site is under the influence of a warm temperate and continental monsoon climate, with cinnamon and brown forest soils developed on limestone. Annual average temperature is 8 °C, with mean monthly minimum temperature of −5 °C in January and mean monthly maximum temperature of 21.5 °C in July^25^. The experimental plots were set up in a mixed *P. tabulaeformis* and *Q. wutaishanica* forest stand, which is a typical zonal vegetation type of the region.

### Experimental design and treatments

The experiment was initiated in September 2009, setting up as a randomized block design with four types of litter placement and three rates of N addition. There were five blocks laid out along the contour of the site and kept at least 1 m apart; within each block, combinations of litter treatment and N addition treatment were randomly assigned to 12 2 × 2 m plots at a minimum of 0.5 m apart^26^. Litter treatments included complete removal of all litter (Litt_nil_), placement of fine woody litter (Litt_woody_), placement of leaf litter (Litt_leaf_), and placement of mixed leaf and fine woody litter (Litt_mix_); N was applied as urea at rates of 0 (N_0_), 5 (N_5_) and 10 gN∙m^−2^∙a^−1^ (N_10_). Each year, all litterfall on Litt_nil_ plots were collected and transferred to Litt_mix_ plots, whilst leaf litter and fine woody litter were sorted and exchanged between Litt_woody_ and Litt_leaf_ plots. N applications were implemented four times per annum, in April, June, August and October, respectively. Urea was used in this study as the form of N addition instead of NH_4_NO_3_ because the latter is not easily obtainable in sufficient quantity in China for security concerns. Previous studies suggested that the form of added N is not of major importance on microbial activity and decomposition^27^,^28^.

### Sample collections and processing

Soil samples were collected in June, August and October of 2014, each to a depth of 0–5 cm on each plot at five random points using a stainless soil corer (3 cm inner diameter and 20 cm long), which were then mixed to form a composite sample. All soil samples were kept in sealed bags before being taken back to laboratory within 2 h, and gravels, roots, and large organic residues were manually removed before passing a 2 mm sieve. Each sample was separated into two parts: one stored at −20 °C for analyses of soil enzyme activities and microbial properties, and the other air-dried and passed through a 0.5-mm sieve for soil physicochemical analyses. Litter samples were collected from forest floor on each plot in April 2015 and oven-dried at 65 °C to constant weight before grounding for determination of mass density and chemical analyses.

### Litter and soil analyses

C concentration in litter samples was measured using the K_2_Cr_2_O_7_-H_2_SO_4_ calefaction method. Litter N concentration was analyzed following the Kjeldahl digestion procedure. Litter AUR was determined as the percentage of residue over litter sample after acidolysis at 105 °C for 2 h by 75% sulfuric acid. SOC was analyzed by K_2_Cr_2_O_7_-H_2_SO_4_ calefaction method. Soil TN was analyzed using the Kjeldahl digestion procedure. Soil MBC and MBN were measured by fumigation-extraction method, using 0.5 M K_2_SO_4_ as extracting agent^29^. Dichromate oxidation method and semi-micro Kjeldahl method were used to determine C and N in the extracts, respectively. Soil microbial activity was determined as the basal rate of microbial respiration (MR), estimated as CO_2_ evolution over a 12-day period of incubation at 25 °C in dark. Soil pH value was measured by mixing the soil sample with deionized water at 1:2.5 ratio (w/v), and the supernatants were measured using a pH meter (HI-9125, Hanna Instruments Inc, Woonsocket, RI).

### Phospholipid fatty acids (PLFA) analysis

Microbial community structure was assessed by extractable ester-linked PLFAs composition analysis^6^,^30^. Concentrations of individual PLFAs were calculated based on 19:0 internal standard concentrations. The indicator PLFAs were used for classification of microbial community types. Bacterial community was represented by PLFAs i14:0, 15:0, i15:0, a15:0, i16:0, 16:1 × 7c, 17:0, a17:0, i17:0, cy17:0, 18:1 × 7c, and cy19:0^31^,^32^. Gram-positive bacteria (G+) are composed of PLFAs i14:0, i15:0, a15:0, i16:0, a17:0, and i17:0^32^,^33^; Gram-negative bacteria (G-) are composed of 16:1ω7c, cy17:0, 18:1ω7c, and cy19:0^34^; actinomycete is composed of 10Me16:0, 10Me17:0, 10Me18:0^31^,^35^,^36^; saprotrophic fungi are represented by 18:2ω6,9c^33^; and arbuscular mycorrhizal fungi (AMF) are represented by 16:1ω5c^33^,^36^,^37^. Other PLFAs such as 14:0, 16:0, 16:1 2OH, 16:1ω9c, 17:1ω8c, and 18:1ω9c were also used for analysis of the microbial composition^32^. The ratio of 18:2ω6,9c to total bacterial PLFAs was used to estimate the ratio of fungal to bacterial biomass (F:B) in soils^31^,^32^. The relative biomass of each microbial composition (their percentages) was calculated by dividing the concentration of each community type by the total amount of all types.

### Enzyme assays

The activities of β-glucosidase (BG; EC: 3.2.1.21) and N-acetyl-β-glucosaminidase (NAG; EC: 3.2.1.30) were determined by *p*-nitrohpenol assays^38^,^39^; and the activities of phenol oxidase (PO) and peroxidase (PER) were measured using DOPA (3,4-Dihydroxy-L-phenylalamine) as their substrates^6^. For PO, the reaction mixture composed of 2 mL 5 mmol/L L-DOPA solution and soil slurry (1 g fresh soil with 1.5 mL 50 mmol/L sodium acetate buffer), and PER activity assays received 2 mL of 5 mmol/L-DOPA and soil slurry (1 g fresh soil with 1.5 mL 50 mmol/L sodium acetate buffer), plus 0.2 mL of 0.3% H_2_O_2_. All total enzyme activities were expressed as μmol g^−1^soil h^−1^. The results of all enzymatic assays were expressed on a dry-weight basis.

### Statistical analyses

All variables were evaluated by analysis of variance (ANOVA) with litter treatment and N addition treatment as main factors for each sampling time. Non-parametric test was conducted if the variances were unequal. The values of BG, NAG, PO, PER, MBC, MBN and MR were also tested by repeated measures analysis of variance (RMANOVA) for the effects of litter treatment and N addition treatment across the three sampling times. Data on soil microbial community structure inferred by PLFAs were tested by permutational multivariate analysis of variance (PERMAVONA). The least significant difference (LSD) test was used to compare means of soil variables in absence of interactive effects between treatments. All data were analyzed using SPSS Version17.0 or R (Version 2.15.3), depending the statistical procedures, with *p* < 0.05 for testing the significance unless otherwise specified.

## Additional Information

**How to cite this article**: Sun, X.-L. *et al.* Soil microbial responses to forest floor litter manipulation and nitrogen addition in a mixed-wood forest of northern China. *Sci. Rep.* **6**, 19536; doi: 10.1038/srep19536 (2016).

## Figures and Tables

**Table 1:** Litter mass density and chemistry in different litter treatment plots in a mixed pine/oak forest in central North China.

Litter treatment	Mass density (kg·m^−2^)	%C	%N	%AUR	Litter C:N	AUR:N
Litt_woody_	1.04 ± 0.11 B	48.8 ± 0.9 A	0.37 ± 0.03 B	40.3 ± 2.0 B	142.0 ± 12.6 A	115.6 ± 11.1 A
Litt_leaf_	2.35 ± 0.29 A	48.7 ± 0.5 A	0.61 ± 0.02 A	49.6 ± 0.9 A	82.1 ± 3.0 B	83.7 ± 2.8 B
Litt_mix_	2.96 ± 0.23 A	47.4 ± 1.0 A	0.64 ± 0.03 A	49.6 ± 1.0 A	79.6 ± 4.5 B	79.5 ± 1.8 B

Treatments began in September 2009 and were applied annually; samples were taken in April 2015. Values presented are means ± standard errors (n = 15). Values designated by the same letters are not significantly different among different litter treatments (*p* < 0.05). Litt_nil_, litter removal (no data presented as measurements are not applicable); Litt_woody_, fine woody litter; Litt_leaf_, leaf litter; Litt_mix_, mixed leaf and fine woody litter.

**Table 2:** Soil organic carbon (SOC), total nitrogen (TN) and C:N ratio under different treatments of litter and nitrogen in a mixed pine/oak forest in central North China.

Treatment		SOC (g·kg^−1^)	TN (g·kg^−1^)	Soil C:N	Soil pH
Litter	Litt_nil_	72.0 ± 3.1 C	4.28 ± 0.13 C	16.5 ± 0.5A	6.61 ± 0.04 A
	Litt_woody_	85.8 ± 4.0 B	4.96 ± 0.16 B	17.2 ± 0.5A	6.58 ± 0.05 A
	Litt_leaf_	104.1 ± 4.8 A	5.73 ± 0.15 A	17.4 ± 0.6A	6.60 ± 0.04 A
	Litt_mix_	101.4 ± 5.5 A	5.47 ± 0.17 A	17.7 ± 0.6A	6.59 ± 0.05 A
N Addition	N_0_	89.2 ± 4.2 A	4.83 ± 0.16 B	17.4 ± 0.5A	6.59 ± 0.04 A
	N_5_	93.6 ± 4.5 A	5.30 ± 0.15 A	17.3 ± 0.4A	6.62 ± 0.04 A
	N_10_	89.6 ± 4.0 A	5.13 ± 0.14 AB	16.9 ± 0.5A	6.58 ± 0.03 A

Treatments began in September 2009 and were applied annually, and samples were collected in June, August and October 2014 and mixed within the same treatment plots. Data are presented as means ± standard errors (n = 5). N_0_, zero rate of nitrogen addition; N_5_, nitrogen addition at 5 g∙m^−2^∙a^−1^; N_10_, nitrogen addition at 10 g∙m^−2^∙a^−1^; Litt_nil_, litter removal; Litt_woody_, fine woody litter; Litt_leaf_, leaf litter; Litt_mix_, mixed leaf and fine woody litter. Values designated by the same letters are not significantly different among nitrogen treatments or among different litter treatments (*p* < 0.05).

**Table 3:** *F*-values and *p*-values (in parentheses) for significance tests of the effects of nitrogen (N) and litter treatments on soil extracellular enzyme activities and microbial variables in a mixed pine/oak forest in central North China.

Variable	Factor	Time of sampling			repeated measurements
		Jun. 2014	Aug. 2014	Oct. 2014	
BG	N	0.024 (0.977)	0.092 (0.912)	2.439 (0.096)	0.398 (0.674)
	Litter	3.335 (0.026)*	8.841 (0.000)***	1.467 (0.233)	4.208 (0.010)*
	N × Litter	0.609 (0.722)	0.775 (0.593)	0.305 (0.931)	0.408 (0.870)
NAG	N	0.214 (0.808)	0.430 (0.652)	*6.217* (0.045)*	1.440 (0.247)
	Litter	*22.24* (0.000)***	*28.07* (0.000)***	5.424 (0.002)**	15.90 (0.000)***
	N × Litter	0.972 (0.455)	0.244 (0.960)	2.306 (0.049)*	1.075 (0.391)
PO	N	0.714 (0.494)	0.011 (0.989)	0.203 (0.817)	0.225 (0.800)
	Litter	*15.21* (0.002)**	8.688 (0.000)***	*8.712* (0.033)*	5.727 (0.002)**
	N × Litter	0.708 (0.645)	0.720 (0.636)	0.137 (0.991)	0.261 (0.952)
PER	N	1.895 (0.160)	0.920 (0.404)	0.587 (0.559)	0.251 (0.779)
	Litter	*3.108* (*0.375)*	3.254 (0.028)*	*6.156* (*0.104)*	3.270 (0.030)*
	N × Litter	0.199 (0.976)	0.419 (0.863)	0.270 (0.948)	0.189 (0.978)
MBC	N	0.086 (0.918)	0.084 (0.919)	2.422 (0.098)	0.569 (0.570)
	Litter	2.561 (0.064)	2.764 (0.050)*	0.507 (0.679)	6.349 (0.001)**
	N × Litter	0.662 (0.681)	0.369 (0.895)	0.535 (0.779)	0.793 (0.580)
MBN	N	0.232 (0.793)	0.144 (0.866)	2.878 (0.064)	0.752 (0.477)
	Litter	3.131 (0.033)*	3.946 (0.013)*	0.587 (0.626)	3.506 (0.022)*
	N × Litter	1.001 (0.436)	0.333 (0.916)	0.303 (0.933)	0.464 (0.832)
MR	N	0.115 (0.891)	0.615 (0.554)	0.073 (0.929)	0.044 (0.957)
	Litter	6.097 (0.001)**	9.435 (0.000)***	8.182 (0.000)***	9.155 (0.000)***
	N × Litter	0.546 (0.771)	0.555 (0.764)	0.908 (0.498)	0.542 (0.774)

Treatments began in September 2009 and were applied annually. BG, β-glucosidase; NAG, N-acetyl-β-glucosaminidase; PO, phenol oxidase; PER, peroxidase; MBC, microbial biomass carbon; MBN, microbial biomass nitrogen; MR, microbial activity. *p < 0.05; **p < 0.01; ***p < 0.001; values in italic are results based on non-parametric tests.
